# Biomarkers of cytokine release syndrome and neurotoxicity related to CAR-T cell therapy

**DOI:** 10.1186/s40364-018-0116-0

**Published:** 2018-01-22

**Authors:** Zhenguang Wang, Weidong Han

**Affiliations:** Molecular & Immunological Department, Bio-therapeutic Department, Chinese PLA General Hospital, No. 28 Fuxing Road, Beijing, 100853 China

**Keywords:** Chimeric antigen receptor, CAR-T, CRS, Neurotoxicity, Biomarker

## Abstract

Severe cytokine release syndrome (CRS) and neurotoxicity following chimeric antigen receptor T cell (CAR-T) therapy can be life-threatening in some cases, and management of those toxicities is still a great challenge for physicians. Researchers hope to understand the pathophysiology of CRS and neurotoxicity, and identify predictive biomarkers that can forecast those toxicities in advance. Some risk factors for severe CRS and/or neurotoxicity including patient and treatment characteristics have been identified in multiple clinical trials of CAR-T cell therapy. Moreover, several groups have identified some predictive biomarkers that are able to determine beforehand which patients may suffer severe CRS and/or neurotoxicity during CAR-T cell therapy, facilitating testing of early intervention strategies for those toxicities. However, further studies are needed to better understand the biology and related risk factors for CRS and/or neurotoxicity, and determine if those identified predictors can be extrapolated to other series. Herein, we review the pathophysiology of CRS and neurotoxicity, and summarize the progress of predictive biomarkers to improve CAR-T cell therapy in cancer.

## Background

In past year, three CD19-directed chimeric antigen receptor T cell (CAR-T) programs, including Novartis’s CTL019, Kite’s KTE-C19, and Juno’s JCAR015, were racing to become the first-ever approved by the US Food and Drug Administration (FDA). Novartis’s CTL019 has been approved recently under the name tisagenlecleucel (KYMRIAH™) by FDA for the treatment of relapsed or refractory (r/r) patients with B-cell acute lymphoblastic leukemia (B-ALL) up to 25 years of age [1]; Kite’s KTE-C19 has also been approved under the name axicabtagene ciloleucel (YESCARTA™) by FDA for the treatment of adult patients with certain types of large B-cell lymphoma who have not responded to or who have relapsed after at least two other kinds of treatment [2]. In contrast, Juno Therapeutics abandoned its CAR-T front-runner JCAR015 after deaths of five patients due to cerebral edema, a neurologic adverse event seen in the pivotal phase II trial(ROCKET) of JCAR015 for adult patients with B-ALL [3]. The extreme consequences of JCAR015 highlights the challenge of how to control the toxicities of CAR-T cell therapy.

In contrast to traditional cancer therapies, CAR-T cells can be considered as ‘living drugs’ which undergo a marked proliferation (100–100,000 fold) in vivo upon antigen engagement [4]. In addition to the potent anti-tumor activity, these CAR-T cells can also give rise to significant side effects. The two most common and concerning toxicities with CAR-T cells are cytokine release syndrome (CRS) and neurotoxicity [5–8]. In the phase II trial of tisangenlecleucel for r/r B-ALL, severe CRS and neurotoxicity were reported in 47% and 15% of patients who received tisangenlecleucel [9]; while in the pivotal trial of axicabtagene ciloleucel for aggressive B-cell non-Hodgkin lymphoma (B-NHL), severe CRS and neurotoxicity occurred in 13% and 28% of patients who received axicabtagene ciloleucel [10]. Guidelines to manage those toxicities with agents that include Interleukin (IL)-6 receptor inhibitor tocilizumab alone or with steroids have been established and incorporated into some of the CAR-T clinical trials [8, 11]. In general, those two toxicities are manageable in most patients, however, it can be life-threatening in some cases, and management of those conditions can be highly challenging [12–15] .

It is critical to understand the pathophysiology of CRS and neurotoxicity for early detection and better management of those conditions. Moreover, it is essential to identify predictive characteristics and biomarkers in patients with severe CRS and neurotoxicity so that it may be possible to risk stratify patients for the development of these complications during CAR-T cell therapy. A few risk factors associated with CRS and neurotoxicity have been identified in the various clinical trials of CAR-T cell therapy conducted so far. Some studies have been able to verify biomarkers that can predict the development and severity of CRS and/or neurotoxicity [13, 14, 16, 17]. Screening patients for high risk of CRS and neurotoxicity can be highly beneficial as these patients can be monitored closely or even be prophylactically treated with preemptive anti-cytokine directed treatment which would effectively mitigate severe CRS and neurotoxicity. In this minireview, we discuss the pathophysiology of CRS and neurotoxicity, and summarize the progress of biomarkers as aids to CAR-T cell therapy in cancer.

## Manifestations of CRS related to CAR-T cell therapy

CRS is a clinical constellation of symptoms including fever, nausea, fatigue, myalgias, malaise, hypotension, hypoxia, coagulopathy and capillary leak, and/or multiorgan toxicity, which has been reported to occur in 30–94% of patients, including grade ≥3 CRS in 1–48% [9, 10, 13, 18–25]. CRS typically occurs 1 to 22 days after CAR-T cell infusion, the median time to onset of CRS is 2–3 days [26, 27].Severe CRS usually starts earlier than the CRS that is not severe [13, 28]. The first clinical sign of CRS in most of patients is high fever, which can even rise to more than 40 °C in some patients [13, 29–31]. Notably, in a large cohort of 133 patients with B-cell malignancies who received anti-CD19 CAR-T cell (JCAR014) following lymphodepletion chemotherapy, the investigators showed that fever in patients with grade ≥ 4 CRS not only presented and peaked earlier after CAR-T cell infusion, but also reached a higher maximum temperature and was of longer duration than that of in patients with grade<4 CRS [13]. Besides the constitutional symptoms such as high fever, myalgias, malaise, some severe cases can experience significant hemodynamic instability and capillary leak with hypotension, tachycardia, hypoxia, tachypnea, hemophagocytic lymphohistiocytosis/macrophage activation syndrome, and or other organ toxicity [8, 11, 32]. Mild-to moderate CRS usually is self-limited, and can be managed with close observation and supportive care. Severe CRS must require intensive medical management with tocilizumab alone or with steroids. Nonetheless, resistant CRS, characterized by no clinical improvement or worsening at any time despite use of tocilizumab and steroids [27, 33], may occur in a small number of patients, and in whom the mortality risk is very high [13, 34, 35]. The time to resolution of CRS is variable, can be ranged from 1 to 60 days after CAR-T cell infusion, and the median time to resolution of CRS is about a week [26, 27]. It must be emphasized is that patients with severe CRS presented delayed hematopoietic recovery [13], potentially increasing the chances of infection, especially in the setting of using tocilizumab that is able to worsen the neutropenia. Empiric antibiotic therapy should be initiated at the beginning of the lymphodepletion chemotherapy.

The cytokine profiles involved in CRS following CAR-T cell therapy encompass not only the effector cytokines such as interferon (IFN)-γ, IL-2, soluble IL-2Rα, IL-6, soluble IL-6R and granulocyte-macrophage colony-stimulating factor (GM-CSF), but also the cytokines mainly secreted by the monocytes and/or macrophages such as IL-1,IL-6, IL-8,IL-10,IL-12, tumor necrosis factor (TNF)-α, IFN-α, monocyte chemotactic protein (MCP)-1, macrophage inflammatory protein (MIP) 1α etc. [36–41]. In addition, some serum biochemical markers, including C-reactive protein (CRP) and ferritin levels are always elevated in patients who experienced CRS after CAR-T cell therapy [13, 28–30, 37, 42, 43]. Monitoring the changes of those laboratory markers after CAR-T cell infusion can give insight in to CRS, and investigational cytokine activation profiles that are associated with CRS have been documented in several CAR-T clinical trials [4, 13, 29]. Of note, the diagnoses, grading and management of CRS are mainly based on abovementioned clinical signs and symptoms rather than those laboratory markers [8, 11, 43, 44].

## Pathophysiology of CRS related to CAR-T cell therapy

CRS is not restricted to CAR-T cell therapy and is associated with therapeutic monoclonal antibodies such as anti-CD3 (OKT3) [45], anti-CD20 (rituximab) [46],anti-CD28 (TGN1412) [47], anti-CD52 (alemtuzumab) [48], CD3/CD19 bispecific antibody (blinatumomab) [49] and anti-PD-1 (nivolumab) [50]. CRS is also known as another similar term: cytokine storm, which highlights the state of the immune system gone awry and inflammatory response flaring out of control [51]. The two terms are used interchangeably in some literature [47, 52, 53], but some researchers use the term cytokine storm to refer to the severe case of CRS. In general, there is a balance between the proinflammatory and anti-inflammatory mechanisms, which determines the intensity of the inflammatory response and maintains the immune homeostasis [51, 54]. The proinflammatory and anti-inflammatory cytokines are regulated by complex regulatory networks involving lymphocytes (B cells, T cells, and/or natural killer cells), myeloid cells (macrophages, dendritic cells, and monocytes) and endothelial cells [55]. Moreover, each cytokine also can exert inductive and inhibitive effects to other cytokines, making a cytokine matrix that is responsible for balance regulation [56]. If this delicate balance ever fails and proinflammatory cytokines prevail, then the outcome may contribute to a cytokine storm.

CRS is a direct result of overproduction of inflammatory cytokines caused by supraphysiological levels of immune activation. However, the detailed mechanism remains poorly defined. Significantly, recent findings in this area may shed light on the underlying mechanism of CAR-T cell induced CRS. (1) CAR is major histocompatibility complex-independent receptor and, thus can function in CD4 and CD8 T cells [57], enabling the biology of CAR-T cells is distinct from that of classic T-lymphocytes. Several preclinical studies have shown that CD4+ CAR-T cells alone have the comparable anti-tumor activity to CD8+ CAR-T cells, and can undergo stronger expansion as well as produce higher amounts of immune-stimulatory cytokines, such as IFN-γ, TNF-α, IL-2 etc. [58, 59]. This unique feature may contribute to the prompt and high cytokine secretion by CAR-T cells upon engagement of the target antigen in either tumor cells or nonmalignant cells. IFN-γ released by the activated CD4+ CAR-T cells and CD8+ CAR-T cells can stimulate macrophages activation and inducing high level of proinflammatory cytokines including IL-12, IL-6, TNF-α,IL-1β, IL-15 and low level of anti-inflammatory cytokines including IL-4,IL-10 to further promote subsequent immune response [60, 61]. IFN-γ–assisted macrophage activation has a direct positive effect on maintaining and enhancing the intensity of the immune response, potentially increasing the likelihood of CRS. The interaction between macrophages and T cells via cytokines may explain why macrophage activation syndrome can occur in a subset of patients who received CAR-T cells [48, 49], and why the cytokine profiles and clinical manifestations of macrophage activation syndrome overlap with those of CRS [50]. (2) Recent two clinical studies have demonstrated that vascular endothelial activation or dysfunction is associated with severe CRS [13, 15]. The data from a study of 133 patients treated with JCAR014 have indicated that biomarkers of endothelial activation including von Willebrand Factor (VWF) and angiopoietin (Ang)-2 are elevated during severe CRS, which is consistent with the presentation of vascular instability, capillary leak, and consumptive coagulopathy in severe CRS [15]. Moreover, the study also has demonstrated that patients with preexisting endothelial activation before infusion of CAR-T cells are prone to develop sever CRS. It is noteworthy that investigators from University of Pennsylvania (Upenn) have confirmed that endothelial cells, in particular blood-vessel endothelial cells, are the key source of IL-6 in CRS by using dual RNA ISH to examination of the IL-6 expression level of lymph node tissue from a patient who succumbed to the CRS after CTL019 infusion [15]. Thus, there may be a possible cascade that progressively amplifies endothelial activation, where, the high concentrations of systemic cytokines such as IFN-γ, IL-6 and TNF-α released by the hyper-activation of CAR-T cells and non-CAR-T cells induce endothelial cell activation, and then the activated endothelial cells produce more IL-6. The high levels of IL-6 may initiate a proinflammatory IL-6-mediated signaling cascade [11], exacerbating the imbalance of proinflammatory and anti-inflammatory cytokines. This observation from Upenn may be informative for elucidating the mechanism of tocilizumab in alleviating CRS.

## Manifestations of neurotoxicity related to CAR-T cell therapy

Neurotoxicity is another prominent toxicity with published reports of 20–64%, including grade ≥3 in 13–52%, and the most common symptoms include encephalopathy, headache, delirium, anxiety, tremor, aphasia; other manifestations of neurotoxicity such as decreased level of consciousness, confusion, seizures and cerebral edema have also been observed in clinical trials of CAR-T cells [5, 9, 10, 14, 18, 19, 22, 30, 62, 63]. The median onset of neurologic events occurs on 4–5 days after CAR-T cell infusion, it can be concurrent with CRS, following resolution of CRS or occur alone [14, 26, 27]. In general, the mild clinical signs are self-limited and resolve within days; more severe symptoms may require supportive care alone or with dexamethasone, and can be complete resolved within 4 weeks. However, some deaths caused by this unexpected toxicity have been documented [14, 21, 64, 65], requiring immediate attention to neurotoxicity management. Similar neurotoxicity has also been reported after administration of blinatumomab [66], a CD3/CD19 bispecific antibody that can result in robust T-cell activation as does the CAR-T cells [67, 68].

## Pathophysiology of neurotoxicity related to CAR-T cell therapy

Thus far, the exact mechanism of CAR-T associated neurotoxicity has not been completely elucidated, and several probable mechanisms may contribute to the development of neurotoxicity. (1) CAR-T cells directly mediate toxicity on central nervous system (CNS) tissues that may have the yet-to-be-detected expression of targeted antigen. To date, the main understanding regarding the CAR-T cell induced neurotoxicity is gained from the trials involving CD19. Multiple anti-CD19 CAR-T cell programs have observed that intravenously infused CAR-T cells can cross the blood-brain barrier (BBB) to a sufficient degree, irrespective of CNS malignancy status at the time of CAR-T cell therapy [28, 29, 34, 42, 69–71]. These CAR-T cells trafficking to cerebrospinal fluid (CSF) are able to eradicate the CNS malignancy efficiently in a subset of patients [42, 70]. However, there is so far no experimental evidence to prove the CD19 expression in CNS tissues. An alternative approach to prove that the CD19 antigen is not involved is using CARs targeting antigens other than CD19, such as CD20 or CD22, and then testing if similar neurotoxicity can be seen in these trials. Recently, Fry and colleges have reported that several patients experience reversible neurologic events including transient visual hallucinations, mild unresponsiveness, mild disorientation, and mild–moderate pain among the first 16 patients with B-ALL who received anti-CD22 CAR-T cells [63]. Davila et al. have documented that no detectable CAR-T cells are found in the CSF of patients who exhibited neurotoxicity [29]. Taken together, these findings provide moderate evidence to disprove this hypothesis; however, the contribution from the direct toxicity effect of CAR-T cells on CNS normal cells expressing targeted antigen cannot be excluded until the conclusive evidence to prove or disprove this hypothesis has been showed. More studies are needed to examine the validity of this hypothesis by using beyond CD19 CAR-T cell products. (2) CNS endothelial cell activation emerges as a driver of CAR-T cell-associated neurotoxicity [72]. Recently, Gust et al. have observed endothelial dysfunction and increased BBB permeability in patients who had neurotoxicity, and constructed a plausible pathophysiologic model to elucidate the development of neurotoxicity; Moreover, they have shown that patients with evidence of endothelial activation before lymphodepletion may be at increased risk of neurotoxicity [14]. In this large cohort of 133 adults with B-cell malignancies(62 B-NHL,47 B-ALL and 24 chronic lymphocytic leukemia) who received lymphodepletion chemotherapy followed by JCAR014, the overall incidence of neurotoxicity is 40%(53 of 133); grade ≥3 neurotoxicity occurs in 21%(28 of 133), including 5 grade 5 neurotoxicity. Gust and colleagues have found that patients who developed grade ≥ 3 neurotoxicity have more severe vascular dysfunction including vascular leak and disseminated inravascular coagulation, which is consistent with widespread endothelial activation evidenced by the elevated serum Ang-2, VWF after CAR-T cell infusion. Moreover, they have also demonstrated that neurotoxicity is associated with early onset of high concentrations of serum cytokines including those that activate endothelial cells, such as IL-6, IFN-γ, and TNF-α. Those high levels of inflammatory cytokines induce endothelial cell activation, resulting in release of Ang-2 and VWF from endothelial Weibel-Palade bodies, and the released VWF binds activated endothelium and sequesters platelets. Increased permeability of the BBB due to the endothelium activation allows transit into CSF of high concentrations of serum cytokines, including IFN-γ and TNF-α, initiating a feed-forward loop of continued endothelial cell and pericyte activation. In the most severe case, this feed-forward loop can cause breakdown of the parenchymal basement membrane and vascular disruption, with cerebral edema, hemorrhage, infarction, and necrosis, and neuronal death as observed in autopsy studies of 2 patients who had fatal neurotoxicity. It must be emphasized that IL-6 may play a crucial role in this feed-forward loop. Significantly, they have shown that an earlier peak of the IL-6 serum concentration is associated with a higher risk of grade ≥ 4 neurotoxicity. In fact, markedly elevated IL-6 levels in CSF and serum of patients experiencing mild encephalopathy with a reversible splenial lesion have been reported [73]. Our group has also observed that IL-6 levels in serum of a patient with B-ALL is highly elevated 1 day after CD19/CD20 bi-specific CAR-T cell infusion, just at the onset of grade 3 neurotoxicity (unpublished data). Furthermore, the National Cancer Institute (NCI) has observed that administration of tocilizumab results in the onset of neurotoxicity in a subset of patients receiving CAR-T cells, which should be attributable to a transient elevated IL-6 level due to the inhibition of IL-6 receptor-mediated clearance by tocilizumab [11]. In short, the work by Gust and colleagues provides the first detailed clinicopathological insights in to the CAR-T cell-associated neurotoxicity, and the confirmation in other CAR-T cell trials is required.

## Risk factors for CRS and neurotoxicity related to CAR-T cell therapy

**Fig. 1:**
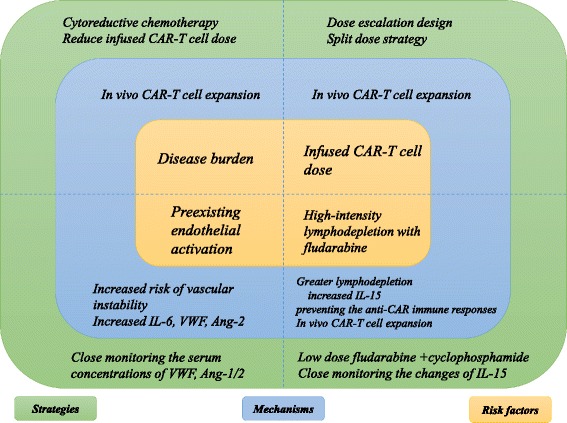
Risk factors for CRS and neurotoxicity. Disease burden and infused CAR-T cell dose have a direct impact on the in vivo CAR-T cell expansion. Enhanced in vivo CAR-T cell expansion also correlates with the high-intensity lymphodepletion with fludarabine, which can result in greater lymphodepletion and prevention of the anti-CAR immune responses. The level of IL-15, one of the cytokines that can improve T cell activation and function, is elevated due to the greater lymphodepletion. Patients with preexisting endothelial cell activation are prone to develop severe CRS and/or neurotoxicity. VWF released by the activated endothelial cell can bind activated endothelium and sequesters platelets. Ang-2, another endothelial cell activation biomarker, can promote the capillary leak. Moreover, activated endothelial cell is a key resource of IL-6 in CRS, and then secreted IL-6 can further facilitate endothelial cell activation, increasing the risk of CRS and/or neurotoxicity. CRS: cytokine release syndrome; CAR-T: chimeric antigen receptor T cell; IL: interleukin; VWF: von Willebrand Factor; Ang:angiopoietin

Based on the above analysis, CRS and neurotoxicity should not be seen as two completely unrelated adverse events, but be proposed as overlapping off-target toxicities resulted from the excessive immune activation either CAR-T cells or non-CAR-T cells. Although neurotoxicity can occur alone in a small number of patients, neurotoxicity severity tracked largely with CRS severity, and both correlated with enhanced CAR-T cell expansion [72]. Any factors that can increase in vivo CAR-T cell numbers, including high disease burden, higher infused CAR-T cell dose, high intensity lymphodepletion regimen, as well as some patient characteristics including preexisting endothelial activation, severe thrombocytopenia may increase the risk of CRS and/or neurotoxicity(Fig. 1). Significantly, as observed in the ROCKET trial [74], patients who have all of these factors or almost all of these factors may be at an extremely high risk of severe CRS and/or neurotoxicity.

### Disease burden

Disease burden is associated with the peak expansion of CAR-T cells in vivo [75, 76], which should be the underlying mechanism of disease burden effect on the occurrence and severity of CRS and neurotoxicity. Disease burden has been a consistent risk factor for CRS following CAR-T cell infusion [19, 28, 29, 42, 75–78], and strong correlation between the severity of CRS and disease burden at the time of CAR-T cell infusion has been noted in multiple clinical trials of CAR-T cell therapy of hematological malignancies [28, 29, 42, 75, 77]. In our phase I trial of CART-20 cells for B-NHL, we have observed that those patients with bulky disease burden defined as the lesions with a maximum diameter greater than 5 cm or more than three lesions have increased risk of CRS [77, 79]. In addition, the association between disease burden and neurotoxicity has also been reported by several programs [14, 16, 80, 81]. For example, Memorial Sloan Kettering Cancer Center (MSKCC) group has identified that the bone marrow leukemic blasts>50% is significantly associated with sever neurotoxicity in the trial of JCAR015 for B-ALL [16].Therefore, debulking therapy such as chemotherapy or radiotherapy to decrease the disease burden is essential to controlling the risk of toxicity following CAR-T cell therapy in the case of high disease burden. A cytoreductive chemotherapy regimen aiming to not only reduce disease burden but also effect a preconditioning regimen has been adopted by our and other groups as an indispensable portion of the CAR-T cell clinical trial protocol [31, 38, 79, 82, 83]. Moreover, using a lower CAR-T cell dose for patients with high disease burden, a strategy called “risk-stratified CAR-T cell dosing” has been successfully implemented by Fred Hutchinson Cancer Research Center (FHCRC) group in a trial of JCAR014 for B-ALL, by which the incidence of neurotoxicity in patients with high disease burden (bone marrow leukemic blasts>20%) is decreased,while the disease response is not impaired [76]. Modifying the CAR-T cell dose seems feasible in B-ALL, but may reduce nodal response in other tumors, and therefore can be proposed as an alternative if necessary [13]. In conclusion, it is a challenge to treat a patient with a high disease burden, and decreasing disease burden before CAR-T cell infusion is worthy of consideration.

### Infused CAR-T cell dose

Although there is no clear dose-toxicity relationship between the number of CAR T cell infused and the occurrence and severity of CRS and/or neurotoxicity, several groups have observed that infused CAR-T cell dose indeed impacts the severity of CRS and/or neurotoxicity [13, 14, 19, 42, 63]. In the abovementioned trial of 133 patients treated with lymphodepletion chemotherapy and JCAR014 at 1 of 3 dose levels (2 × 10^5^, 2 × 10^6^ or 2 × 10^7^ JCAR014 cells/kg), six of seven patients who developed grade ≥ 4 neurotoxicity were treated during the dose-escalation phase of the protocol and received JCAR014 at a dose level of 2 × 10^7^ JCAR014 cells/kg, while only 1 of 79 patients treated after completion of the CAR-T cell dose-escalation phase and receiving JCAR014 at a dose level of 2 × 10^6^ JCAR014 cells/kg developed grade ≥ 4 neurotoxicity [14]. This remarkable effect of infused CAR-T cell dose on the severity of toxicity highlights the necessity of the dose escalation design when testing a new CAR-T cell product.

### High-intensity lymphodepletion with fludarabine

Turtle and colleagues have reproted that addition of fludarabine (Flu) to cyclophosphamide (Cy) in lymphodepletion enhances in vivo CAR-T cell expansion, which may be attribute to greater lymphodepletion and delaying or preventing the anti-CAR immune responses [76, 84]. Greater lymphodepletion can lead to increased levels of homeostatic cytokines, including IL-15, one of the cytokines that support T cell proliferation and enhance T-cell function [85–87]. Kochenderfe et al. have documented that serum IL-15 levels of all 22 patients are elevated after lymphodepletion chemotherapy and are strongly associated with the peak levels of CAR-T cells in vivo, and associated with the neurotoxicity [88]. Similar to that reported by Kochenderfe, Gilbert has also demonstrated that early and rapid CAR- T cell expansion and a rise in IL-15 levels are primary contributors to the high rates of cerebral edema seen with JCAR015 in the phase II ROCKET trial. Moreover, he has noted that the early and rapid expansion of CAR-T cells appears to correlate with higher levels of IL-15, which are increased before commencing CAR-T cell infusion due to the combined use of Flu and Cy [74]. Together, it is not surprising that addition of Flu at any given CAR-T cell dose will increase the risk of CRS and neurotoxicity, as observed by investigators from FHCRC in the larger cohort of 133 patients treated with lymphodepletion followed by JCAR014 infusion [13, 14].

### Preexisting endothelial activation

As discussed above, patients with preexisting endothelial activation before infusion of CAR-T cells are at higher risk of developing sever CRS and/or neurotoxicity, suggesting that preexisting endothelial activation might be a previously unrecognized risk factor for severe CRS and/or neurotoxicity [13, 14]. Notably, severe thrombocytopenia before lymphodepletion chemotherapy is associated with higher risk of developing CRS [13]. MSKCC group have showed that low platelet (platelet<60) is significantly correlated with severe neurotoxicity [16]. These findings imply that patients with severe thrombocytopenia might be prone to endothelial activation given the fact that platelets are one of the few sources of the endothelial stabilizing cytokine, Ang-1 [13].Thus, close examination of endothelial activation biomarkers such as VWF, Ang-2 before and after CAR-T cell infusion should be recommended, which may make sense for clinical management during CAR-T cell therapy. However, more studies are required to further explore the association between preexisting endothelial activation and higher risk of CRS and/or neurotoxicity.

### Other risk factors

Preexisting neurologic comorbidities may be a risk factor for neurotoxicity [14]. Moreover, neurotoxicity is more frequent in younger patients [14, 74] and non-heavily pretreated patients [74]; a plausible reason this phenomenon may be that the expansion ability of CAR-T cells from those patients is not compromised.

## Predictive biomarkers of CRS and neurotoxicity related to CAR-T cell therapy

An area of ongoing research is whether the early changes of cytokine profiles or serum biochemical markers can be applied to forecast severity of CRS and/or neurotoxicity, so as to guiding the preemptive anti-cytokine directed treatment. It is plausible that serum cytokine levels serve as biomarkers due to the natural feature of CRS characterized by significant systemic inflammation with elevated inflammatory cytokines [11].Positive significant correlation between serum cytokine levels after CAR-T cell infusion and severity of CRS has been identified across different institutions [4, 80, 89]. Recently, Teachey and colleagues have reported that they could accurately predict which patients would develop severe CRS with the forward-selected logistic regression model including three cytokines. Particularly in the pediatric patients, the modeling analyses based on evaluating the IFN-γ, IL-13, MIP1α concentrations within the 72 h of infusion are highly accurate with sensitivity of 100% and specificity of 96%, and are validated in an independent cohort of 12 pediatric patients [4]. Their work highlights that an optimal predictive biomarker for CRS must meet the following requirements: (1) significant association is not enough; it must be able to predict the onset of severe CRS with a high sensitivity and specificity. For instance, CRP and ferritin are associated with CRS, but both of these biomarkers fail to predict development of severe CRS [4]; (2) evaluating cytokines must be early. 72 h appears longer, given severe CRS can occur 24 to 72 h after CAR-T cell infusion in a subset of patients. In contrast to the model constructed by Teachey and colleagues, investigators from FHCRC have developed an more simple and timely classification tree modeling including fever and single cytokine for earlier identification of patients at high risk of grade ≥ 4 CRS. The best sensitivity and specificity are obtained by testing serum MCP-1 in patients with fever ≥38.9 °C within 36 h of infusion [13]; Moreover, by combination of IL-6 ≥ 16 pg/mL in the first 36 h after CAR-T cell infusion, this algorithm can be used as a predictive biomarker for grade ≥ 4 neurotoxicity with sensitivity of 100% and specificity of 94% [14]. Of note, MSKCC group has demonstrated that some baseline characteristics of patients can be proposed as a predictive biomarker for severe neurotoxicity with 95% sensitivity and 70% specificity, including baseline blood platelet level<60 or mean corpuscular hemoglobin concentration>33.2% and morphologic disease (>5% blasts) [16]. It is not yet clear if these predictive models will be generalizable, confirmation is required in further studies. Moreover, it is a challenge for testing for cytokines such as MCP-1, MIP1α that cannot be readily available in most clinical hospitals currently [11]. Therefore, modeling using combination of clinical parameters may be more widely used in practice.

## How to decrease the CRS and neurotoxicity related to CAR-T cell therapy

Strategies for decreasing CRS and/or neurotoxicity fall into two categories, prevention strategy aiming to lessen the occurrence of severe toxicities, and remedy strategy in an effort to minimize the toxicity once the fatal toxicities associated to CAR-T cells occur.

Based on the risk factors described above, prevention strategies mainly encompass using debulking chemotherapy to reduce the disease burden prior CAR-T cell infusion, and reducing the CAR-T cell dose in patients with high disease burden, particularly in the patients with B-ALL. More importantly, intervention early in those patients at highest risk identified by close monitoring those predictive biomarkers. Currently, in the case of management of severe or life-threatening CRS occurring after CAR-T cell therapy, tocilizumab 8 mg/kg I.V (maximum dosage per infusion does not exceed 800 mg) is recommended [8].This recommendation may be also suitable for the early intervention with CRS, as evidenced by the observation by the Seattle group that early intervention with tocilizumab ±dexamethasone appears to decrease the rates of severe CRS while not jeopardizing the efficacy [90]. Of note, at the 2017 annual meeting of the American Society of Hematology, Locke and colleges have presented that prophylactic 8 mg/kg of tocilizumab on day 2 post KTE-C19 infusion plus 750 mg of levetiracetam twice a day on day 0 reduce the incidence of severe CRS but not neurotoxicity in a safety expansion cohort of ZUMA-1, providing a preliminary supporting evidence for prophylactic tocilizumab to lessen the occurrence of severe CRS [91]. However, it seems that prophylactic or early tocilizumab has not beneficial effect on neurotoxicity, highlighting the necessity of development of preemptive therapies rather than tocilizumab for management of neurotoxicity.

Best-in-class example of remedy strategy is the addition of “suicide” or “elimination” genes in to CAR-T cells, enabling the selective depletion of CAR-T cells in the event that severe toxicity occurs [92, 93]. Inducible caspase-9 (iCasp9) enzyme can be activated and leads to the rapid death of T cells expressing it when exposed to a synthetic dimerizing drug AP1903 [94], and several clinical trials evaluating iCasp9-modified CAR-T cells are enrolling patients (NCT02274584 and NCT02414269) [92]. However, the dimerizing drug AP1903/Rimiducid cannot be available in china, potentially limiting the widespread use of this suicide system in CAR-T cells. This selective depletion can also be mediated by the clinically approved therapeutic antibody(cetuximab or rituximab) when the transduced cells are engineered to express the antibody targeted cell surface antigen such as truncated EGFR (tEGFR) [95] or RQR8(combining target epitopes from both CD34 and CD20 antigens) [96].tEGFR has been used by Juno therapeutics in its anti-CD19 CAR-T cell products including JCAR014 and JCAR017 [19, 76], while RQR8 has been incorporated by Celletics company in to its universal anti-CD19 CAR-T cell product [97]. Nonetheless, it is a concern whether this cell ablation through antibody-dependent cellular cytotoxicity can rapidly start in case of severe toxicity.

## Conclusions

CRS and neurotoxicity are two potentially life-threatening complications of CAR-T cell therapy, and a gradually growing body of research supports that both of these two toxicities are associated with the enhanced in vivo CAR-T cell expansion, implying the pathophysiology of these two distinct clinical syndromes is intertwined. Endothelial cell activation radically expands our understanding of CRS and neurotoxicity. Some simple and timely predictive biomarkers such as the combination of fever and MCP-1 make sense of early intervention for the patients at high risk of CRS and/or neurotoxicity, but confirmation is required in further clinical studies. The optimal pre-emptive therapy of high-risk patients is unknown. Prophylactic or early tocilizumab seems to benefit CRS but not neurotoxicity; endothelial stabilizing agents may be effective in neurotoxicity. Systematic investigations are necessary to determine whether those early interventions affect the anti-tumor activity of CAR-T cells. Efforts to further elucidate the pathologic characteristics of thes two toxicities, identification of related biomarkers, and optimize management strategies for those syndromes will make great sense to safely deliver CAR-T cell therapy.

## Abbreviations

Ang: Angiopoietin
B-ALL: B cell acute lymphoblastic leukemia
BBB: Blood–brain barrier
B-NHL: B-cell non-Hodgkin lymphoma
CAR-T: Chimeric antigen receptor T cell
CNS: Central nervous system
CRP: C-reactive protein
CRS: Cytokine release syndrome
CSF: Cerebrospinal fluid
Cy: Cyclophosphamide
FDA: US Food and Drug Administration
FHCRC: Fred Hutchinson Cancer Research Center
Flu: Fludarabine
GM-CSF: Granulocyte-macrophage colony-stimulating factor
iCasp9: Inducible caspase-9
IFN: Interferon
IL: Interleukin
MCP: Monocyte chemotactic protein
MIP: Macrophage inflammatory protein
MSKCC: Memorial Sloan Kettering cancer center
NCI: National Cancer Institute
r/r: Relapsed or refractory
TNF: Tumor necrosis factor
Upenn: University of Pennsylvania
VWF: Von Willebrand factor
